# Dietary intake of fish and n-3 polyunsaturated fatty acids and risk of postpartum depression: a nationwide longitudinal study – the Japan Environment and Children's Study (JECS)

**DOI:** 10.1017/S0033291719002587

**Published:** 2020-10

**Authors:** Kei Hamazaki, Kenta Matsumura, Akiko Tsuchida, Haruka Kasamatsu, Tomomi Tanaka, Mika Ito, Hidekuni Inadera, the Japan Environment and Children's Study Group

**Affiliations:** 1 Department of Public Health, Faculty of Medicine, University of Toyama, Toyama, Japan; 2 Toyama Regional Center for JECS, University of Toyama, Toyama, Japan; 3 Department of Pediatrics, Faculty of Medicine, University of Toyama, Toyama, Japan; 4 Department of Obstetrics and Gynecology, Faculty of Medicine, University of Toyama, Toyama, Japan

**Keywords:** Fish intake, n-3 polyunsaturated fatty acids, postpartum depression, pregnancy, serious mental illness

## Abstract

**Background:**

Pregnant women require increased levels of n-3 polyunsaturated fatty acids (PUFAs) due to the demands of the growing fetus. Although some evidence indicates that maternal intake of fish and n-3 PUFAs is associated with reduced risk of postpartum depression, the results are inconsistent.

**Methods:**

We investigated whether dietary consumption of fish and/or n-3 PUFAs during pregnancy is associated with a reduced risk of maternal postpartum depression at 6 months after delivery and of serious mental illness at 1 year in a Japanese population. After exclusion and multiple imputation from a dataset comprising 103 062 pregnancies obtained in the Japan Environment and Children's Study, we evaluated 84 181 and 81 924 women at 6 months and 1 year after delivery, respectively.

**Results:**

Multivariable logistic regression showed a reduced risk of postpartum depression at 6 months in the second to fifth quintiles *v*. the lowest quintile for fish and n-3 PUFA intake, with trend tests also revealing a significant linear association. At 1 year after delivery, fish intake was associated with a reduced risk of serious mental illness in the second to fifth quintiles *v*. the lowest quintile for fish and in the third to fifth quintiles *v*. the lowest quintile for n-3 PUFA intake, with trend tests also revealing a significant linear association.

**Conclusions:**

Women with higher fish and/or n-3 PUFA intake showed reduced risk of postpartum depression at 6 months after delivery and of serious mental illness at 1 year after delivery.

## Introduction

In an ecological study conducted in 22 countries, Hibbeln (2002) was the first to show a negative association between fish consumption and the prevalence of postnatal depression. Since this finding, many randomized control trials (RCTs) and observational studies have been reported. The first RCT, conducted in the United States with lactating women who received a relatively low dose of pure docosahexaenoic acid (DHA; 200 mg/day) for 17 weeks, revealed no significant beneficial effect on postpartum depression (Llorente *et al*., 2003). The first RCT in Asia, which involved a relatively high dose of n-3 polyunsaturated fatty acids (PUFAs) [2.2 g eicosapentaenoic acid (EPA) + 1.2 g DHA/day], was conducted by Su *et al*. (2008) in Taiwan with pregnant women who had major depressive disorder [according to Diagnostic and Statistical Manual of Mental Disorders (DSM), Fourth Edition criteria] and found therapeutic benefits of n-3 PUFAs. The largest RCT (*n*  =  2399), conducted in Australia with pregnant women using fish oil (DHA 800 mg + EPA 100 mg/day), showed no therapeutic benefits of n-3 PUFAs (Makrides *et al*., 2010). A recent RCT, conducted in collaboration between Japan and Taiwan with 108 pregnant women who had depressive symptoms and involving a moderate dose of n-3 PUFAs (1.2 g of EPA + 0.6 g of DHA/day), revealed no significant effect of n-3 PUFA supplementation (Nishi *et al*., 2019). Although the results are still inconsistent, a meta-analysis investigating the effect of n-3 fatty acids as monotherapy for acute major depressive disorders in pregnant women (Wei-Hong *et al*., 2017) found that n-3 fatty acids were more efficacious than placebo.

As for the observational studies, a very large study involving 9960 pregnant women in the United Kingdom revealed that lower maternal intake of n-3 PUFAs assessed at 32 weeks of gestation was associated with a concurrent high level of depressive symptoms (Golding *et al*., 2009). Weak associations were also found between n-3 PUFA intake assessed at 32 weeks of gestation and depression assessed at 18 weeks of gestation and at 8 months (but not 2 months) after delivery (Golding *et al*., 2009). Another large observational study, conducted in Denmark with 54 202 pregnant women, revealed no association of fish and/or n-3 PUFA intake in mid-pregnancy with risk of hospital admission for postpartum depression (Strøm *et al*., 2009). However, intake of fish, but not n-3 PUFAs, was associated with risk of being prescribed an antidepressant. Two cohort studies (Miyake *et al*., 2006; Kobayashi *et al*., 2017) have measured postpartum depression beyond 1 month after delivery in Japan. One study found inverted J-shaped relationships between the intake of n-3 PUFAs and DHA and the risk of postpartum depression; adjusted odds ratios (ORs) comparing the middle quintile with the lowest quartile showed borderline significance (*p*  =  0.06 and 0.10, respectively) (Miyake *et al*., 2006). The other study found no significant associations of EPA, DHA, and n-3 PUFA intakes in late pregnancy with postpartum depression at either 1 or 6 months after delivery (Kobayashi *et al*., 2017). A meta-analysis comparing the levels of PUFA indices between women with perinatal depression and healthy controls revealed that those with perinatal depression had significantly lower total n-3 PUFA and DHA levels and significantly increased n-6/n-3 ratios (Lin *et al*., 2017).

In our recent large cohort study conducted with women during and after pregnancy in Japan (*n*  =  75 139–79 346), multivariable logistic regression analysis revealed that intake of fish, but not n-3 PUFAs, showed some degree of association with reduced risk of psychological distress during early pregnancy (Hamazaki *et al*., 2018). During mid-late pregnancy, the associations strengthened for both fish and n-3 PUFA intake, with a slightly weaker association after pregnancy for postpartum depression. The associations were weaker for n-3 PUFA intake than for fish intake.

The prevalence of postpartum depression peaks at about 1 month (Ishikawa *et al*., 2011; Shimizu *et al*., 2015; Iwata *et al*., 2016), but its symptoms become fixed and intractable at about 6 months (Denckla *et al*., 2018). Despite conventional thinking that 6 months is outside the standard postpartum depression period (American Psychiatric Association, 2013), many experts believe that the postpartum depression period should be extended to as long as 12 months (Gaynes *et al*., 2005). Accordingly, in the present study, we extended the period of postpartum depression to 6 months and that of serious mental illness to 1 year to more comprehensively determine if these conditions are related to fish and/or n-3 PUFA intake.

## Methods

### Study population

The Japan Environment and Children's Study (JECS) protocol has been described elsewhere (Kawamoto *et al*., 2014; Michikawa *et al*., 2018). Briefly, the aim of the JECS, a nationwide government-funded birth cohort study, is to evaluate the impact of certain environmental factors on child health and development. The pregnant participants in the JECS were enrolled from 15 Japanese regions from January 2011 to March 2014 (Kawamoto *et al*., 2014; Michikawa *et al*., 2018). The eligibility criteria for participants (expectant mothers) are as follows (Kawamoto *et al*., 2014): (1) residing in the study areas at the time of recruitment and expecting to reside continuously in Japan for the foreseeable future; (2) having an expected delivery date between 1 August 2011 and mid-2014; and (3) participating in the study without difficulty, that is, able to understand the Japanese language and complete a self-administered questionnaire. Those residing outside the study areas were excluded from the study even if they visited cooperating healthcare providers working within the study areas. The sample size was determined in advance to ensure sufficient statistical power for rare diseases with ⩽1% prevalence.

**Fig. 1.**
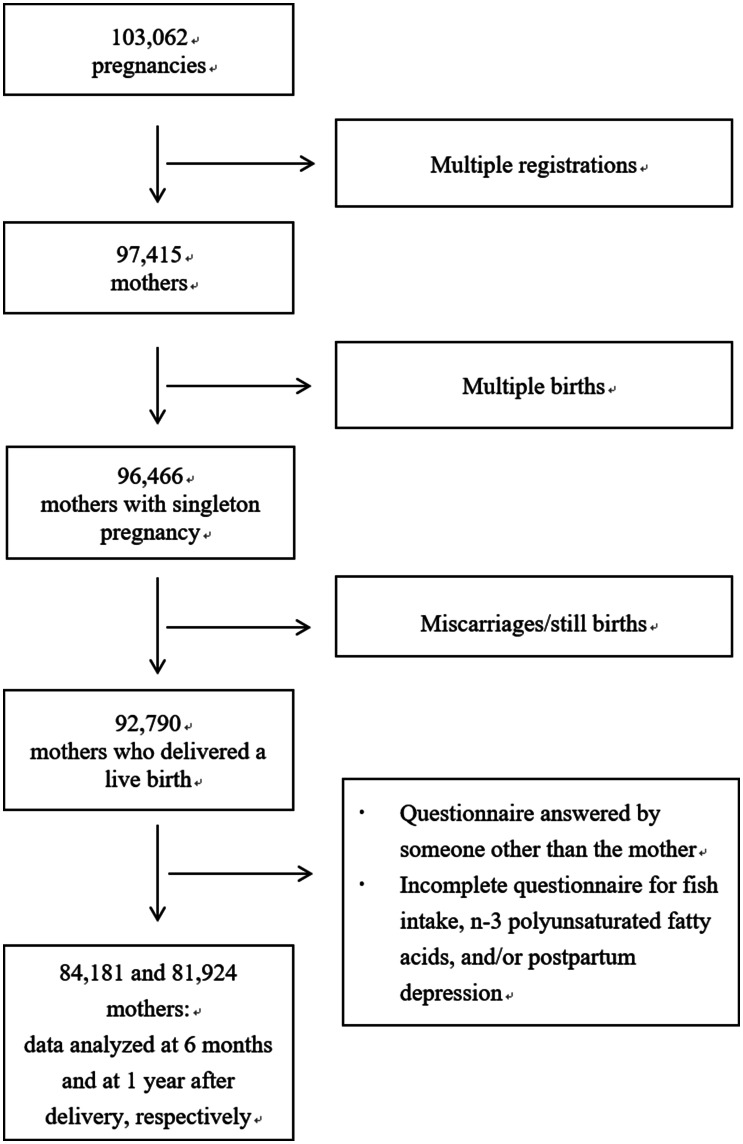
Flow diagram of the recruitment and exclusion process for the participants.

The present study uses data from the jecs-an-20180131 dataset, which was released in March 2018. The full dataset comprises 103 062 pregnancies, but 5647 multiple registrations, 949 multiple births, and 3676 miscarriages/still births were excluded (Fig. 1). Among 92 790 mothers who delivered a live birth, we excluded the following: 160 whose 6-month questionnaires were filled out by someone else (142 by fathers, 14 by grandmothers, and 4 by others) and 6532 whose questionnaires were missing data on who filled them out. The same occurred for 258 1-year questionnaires (227 completed by fathers, 25 by grandmothers, and 6 by others) and 9239 questionnaires missing data on who filled them out. After exclusion of incomplete questionnaire for fish intake, n-3 PUFAs, and/or postpartum depression, there were 84 181 and 81 924 mothers for the 6-month and 1-year analyses, respectively, and multiple imputations were conducted for the missing values for covariates. Imputations were performed using chained equations (van Buuren, 2007) to obtain five imputed datasets. Data were imputed differently according to time point (i.e. at age 6 months and 1 year). Auxiliary variables related to covariates were included to preserve the assumption of ‘missing at random’.

Approval for the study protocol was granted by the Japanese Ministry of the Environment's Institutional Review Board on Epidemiological Studies and the ethics committees of all participating institutions. All participants provided written informed consent.

### Measurements of fish and PUFA intakes

Dietary consumption of fish and total n-3 PUFAs was determined by the Food Frequency Questionnaire, which is semi-quantitative and has been validated for use in large-scale Japanese epidemiologic studies (Yokoyama *et al*., 2016). It contains 171 food and beverage items, including 21 items of fish or shellfish. The JECS dataset does not include data on individual fatty acid subtypes. Participants were asked how often they consumed each food type during mid-late pregnancy (covering dietary intake after the participant learned of the pregnancy). The standard portion size for each food type was categorized as small (50% smaller than standard), medium (same as standard), or large (50% larger than standard). Nine frequency categories for each item were used: less than 1 time/month, 1–3 times/month, 1–2 times/week, 3–4 times/week, 5–6 times/week, every day, 2–3 times/day, 4–6 times/day, and ⩾7 times/day. We calculated the daily intake of fish (g/day) by multiplying the frequency of consumption by the standard portion size for each fish item. We used the fatty acid composition table of Japanese foods (Ministry of Education, Culture, Sports, Science, and Technology, 2010) to calculate the daily intake of n-3 PUFAs. We performed log-transformation of fish and n-3 PUFA intake and calculated the energy-adjusted intake using the residual model (Willett *et al*., 1997).

### Assessment of postnatal depression and serious mental illness

The Kessler Psychological Distress Scale (K6) was administered at 1 year after delivery. The K6 was developed by Kessler *et al*. (2002) and the Japanese version was validated by Furukawa *et al*. (2008). The K6 comprises six items rated on a 5-point scale (0–4). The total scores range from 0 to 24, with higher scores indicating a greater degree of psychological distress. ‘Serious mental illness’ is defined as K6 score ⩾13; this value has been suggested as the optimal cut-off to balance false-positive and false-negative results (Kessler *et al*., 2003). Furthermore, this cut-off is widely used in other epidemiological studies (Kessler *et al*., 2008; Lee *et al*., 2012; Yoshida *et al*., 2016). The Edinburgh Postnatal Depression Scale (EPDS) was used to assess postpartum depression at 6 months after delivery. The EPDS consists of 10 items rated on a 4-point scale (0–3) (Cox *et al*., 1987). We defined an EPDS score ⩾9 as indicating postpartum depression; this has been suggested as the optimal cut-off for the Japanese population, and its validity and reliability have been reported elsewhere (Okano *et al*., 1996).

### Statistical analysis

Data are expressed as the mean ± standard deviation or median unless stated otherwise. To estimate the risks of serious mental illness and postpartum depression for each level of fish intake and n-3 PUFA intake, we categorized the participants according to quintile of fish or n-3 PUFA intake. We then performed logistic regression analysis to calculate ORs and 95% confidence intervals (CIs). In tests for trends, the median intakes of each category were assigned to the quintile distributions for fish intake and n-3 PUFA intake and were evaluated as continuous variables. For an exploratory study of the lower threshold for an effect of n-3 PUFAs and fish on the risk of postpartum depression at 6 months and of serious mental illness at 1 year, we created the lowest decile by splitting the lowest quintile in half and using the lowest decile as a reference.

The confounding factors and covariates considered in this study were as follows: age; previous deliveries (primiparous or multiparous); body mass index at 1 month after delivery (<18.5, 18.5–25, or ⩾25 kg/m^2^); highest maternal educational level (1, junior high or high school; 2, technical junior college, technical/vocational college, or associate degree; or 3, bachelor's or postgraduate degree); annual household income (<4 million, 4–6 million, or >6 million JPY); marital status at 6 months after delivery [1, married (including common-law status); 2, divorced (including elimination of common-law status); or 3, widowed or other]; alcohol intake at 1 month after delivery (1, never; 2, previously drank alcohol but quit; 3, 1–3 times per month; or 4, ⩾1 time per week); smoking status at 1 month after delivery (1, never smoked; 2, previously smoked but quit before learning of current pregnancy; 3, previously smoked but quit after learning of current pregnancy; or 4, currently smoking); physical activity during mid-late pregnancy [MET·min/day (metabolic equivalent of a task measured as the number of minutes per day)]; employment status during mid-late pregnancy for the analysis of EPDS (yes or no); employment status at 1 year after delivery for the analysis of serious mental illness (yes or no); history of anxiety disorder (yes or no); history of depression (yes or no); any sadness experienced during the past year (yes or no) (multiple answers allowed: parent's death, partner's death, child's death, parent's illness or injury, child's illness or injury, partner's illness or injury, own illness or injury, partner's dismissal, own dismissal, close relative or friend's death, considerable debt, change in family structure such as grandparent(s) moving in, divorce, moving house, marital problems, and others); use of EPA and/or DHA supplementation (yes or no); and presence of any congenital anomaly (yes or no). Two-sided *p* values less than 0.05 were considered to indicate statistical significance. Data were analyzed using SAS version 9.4 software (SAS Institute Inc., Cary, NC).

## Results

Table 1 shows maternal characteristics according to quintile for fish consumption, which was adjusted for energy intake using the residual method, and online Supplementary Table S1 shows maternal characteristics according to quintile for n-3 PUFA consumption, which was also adjusted for energy intake. Compared with women who reported low-fish intake, women with higher fish intake were slightly older and were more likely to be multiparous, a nonsmoker, and have a higher level of education and higher annual household income. Dietary n-3 PUFA intake showed very similar associations (online Supplementary Table S1).

**Table 1.** Characteristics according to quintiles for fish intake during pregnancy in women (*n*  =  84 181)

	Quintile for fish intake				
	1 (low)	2	3	4	5 (high)
Median intake of fish^a^, g/day	5.2	18.3	29.7	43.3	69.3
Age at delivery, years	30.5	31.2	31.5	31.7	31.6
Previous deliveries, *n* (%)					
Primipara	8160 (48.5)	7237 (43.0)	7021 (41.7)	6944 (41.3)	7161 (42.5)
Multipara	8676 (51.5)	9599 (57.0)	9816 (58.3)	9892 (58.8)	9675 (57.5)
BMI at one month after delivery, *n* (%)					
<18.5	770 (4.6)	805 (4.8)	805 (4.8)	877 (5.2)	1004 (6.0)
18.5–<25	13 243 (78.7)	13 364 (79.4)	13 472 (80.0)	13 459 (79.9)	13 265 (78.8)
⩾25	2823 (16.8)	2668 (15.9)	2561 (15.2)	2500 (14.9)	2567 (15.3)
Highest educational level, *n* (%)					
Junior high school or high school	6974 (41.4)	5876 (34.9)	5574 (33.1)	5322 (31.6)	5777 (34.3)
Technical junior college, technical/vocational college or associate degree	6949 (41.3)	7318 (43.5)	7298 (43.4)	7274 (43.2)	7056 (41.9)
Bachelor's degree, postgraduate degree	2913 (17.3)	3642 (21.6)	3965 (23.6)	4240 (25.2)	4004 (23.8)
Annual household income (JPY), *n* (%)					
<4 million	7823 (46.5)	6765 (40.2)	6523 (38.7)	6111 (36.3)	6254 (37.2)
4–6 million	5216 (31.0)	5590 (33.2)	5671 (33.7)	5712 (33.9)	5728 (34.0)
>6 million	3797 (22.6)	4481 (26.6)	4643 (27.6)	5014 (29.8)	4854 (28.8)
Marital status, *n* (%)					
Married (including common law marriage)	16 356 (97.2)	16 560 (98.4)	16 572 (98.4)	16 575 (98.5)	16 517 (98.1)
Divorced	201 (1.2)	119 (0.7)	113 (0.7)	121 (0.7)	142 (0.8)
Widowed others	280 (1.7)	157 (0.9)	152 (0.9)	140 (0.8)	177 (1.1)
Alcohol intake, *n* (%)					
Never	15 360 (91.2)	15 448 (91.8)	15 458 (91.8)	15 391 (91.4)	15 367 (91.3)
Ex-drinker	764 (4.5)	701 (4.2)	754 (4.5)	791 (4.7)	758 (4.5)
1–3 times/month	477 (2.8)	454 (2.7)	428 (2.5)	449 (2.7)	474 (2.8)
≧1 or more	234 (1.4)	232 (1.4)	197 (1.2)	205 (1.2)	237 (1.4)
Smoking status, *n* (%)					
Never	9028 (53.6)	9924 (58.9)	10 232 (60.8)	10 435 (62.0)	10 204 (60.6)
Did previously but quit before learning of pregnancy	3848 (22.9)	3909 (23.2)	3737 (22.2)	3748 (22.3)	3749 (22.3)
Did previously but quit after learning of pregnancy	3048 (18.1)	2395 (14.2)	2273 (13.5)	2139 (12.7)	2268 (13.5)
Currently smoking	911 (5.4)	608 (3.6)	594 (3.5)	513 (3.1)	614 (3.7)
Median physical activity (mets·min/day)	70.7	70.7	70.7	70.7	70.7
History of anxiety disorder, yes (%)	574 (3.4)	480 (2.9)	447 (2.7)	441 (2.6)	464 (2.8)
History of depression, yes (%)	579 (3.4)	505 (3.0)	495 (2.9)	480 (2.9)	537 (3.2)
Experiencing sadness during the past year	7397 (43.9)	7566 (44.9)	7494 (44.5)	7519 (44.7)	7382 (43.9)
Employed, *n* (%)	9539 (56.7)	9229 (54.8)	9148 (54.3)	9022 (53.6)	8868 (52.7)
Use of EPA and/or DHA supplementation, yes (%)	548 (3.3)	503 (3.0)	513 (3.1)	486 (2.9)	561 (3.3)
Any congenital anomaly, *n* (%)	380 (2.3)	402 (2.4)	348 (2.1)	372 (2.2)	396 (2.4)

BMI, body mass index.

a Dietary intake during pregnancy (after learning of pregnancy).

During follow-up, 11.6% of women (9761/84 181) had postpartum depression (EPDS ⩾ 9) at 6 months after delivery and 2.6% (2127/81 924) had serious mental illness (K6 ⩾ 13) at 1 year after delivery. Table 2 shows the multivariable ORs and 95% CIs for postpartum depression at 6 months after delivery and serious mental illness at 1 year after delivery according to quintile for fish and n-3 PUFA intake during pregnancy. Multivariable logistic regression showed a reduced risk of postpartum depression at 6 months in the second to fifth quintiles *v*. the lowest quintile for fish and n-3 PUFA intake, with trend tests also revealing a significant linear association. At 1 year after delivery, fish intake was associated with a reduced risk of serious mental illness in the second to fifth quintiles *v.* the lowest quintile for fish and in the third to fifth quintiles *v.* the lowest quintile for n-3 PUFA intake, with trend tests also revealing a significant linear association.

**Table 2.** ORs (95% CIs) for postpartum depression at 6 months and postpartum severe mental illness at 1 year after delivery according to quintiles for fish or n-3 PUFA intake during pregnancy

Variable	Median intake, g/day^a^	Prevalence, case/total (%)	Crude OR (95% CI)	Adjusted OR (95% CI)^b^
*Postpartum depression at 6 months after delivery (n* = *84* *181)*				
Fish				
Q1	5.2	2319/16 836 (13.8)	1.00	1.00
Q2	18.3	1977/16 836 (11.7)	**0.83** (**0.78–0.89**)	**0.90** (**0.84–0.96)**
Q3	29.7	1833/16 837 (10.9)	**0.77 (0.72–0.82)**	**0.85 (0.79–0.91)**
Q4	43.3	1805/16 836 (10.7)	**0.75 (0.70–0.80)**	**0.85 (0.79–0.91)**
Q5	69.3	1827/16 836 (10.9)	**0.76 (0.71–0.81)**	**0.84 (0.78–0.90)**
*p* for trend			**<0.0001**	**<0.0001**
n-3 PUFAs				
Q1	1.09	2310/16 836 (13.7)	1.00	1.00
Q2	1.46	1999/16 836 (11.9)	**0.85 (0.80–0.90)**	**0.88 (0.83–0.94)**
Q3	1.74	1839/16 837 (10.9)	**0.77 (0.72–0.82)**	**0.82 (0.77–0.88)**
Q4	2.04	1715/16 836 (10.2)	**0.71 (0.67–0.76)**	**0.76 (0.71–0.82)**
Q5	2.57	1898/16 836 (11.3)	**0.80 (0.75–0.85)**	**0.84 (0.78–0.89)**
*p* for trend			**<0.0001**	**<0.0001**
*Postpartum psychological distress at 1 year after delivery (n* = *81* *924)*				
Fish				
Q1	5.3	580/16 384 (3.5)	1.00	1.00
Q2	18.4	452/16 385 (2.8)	**0.77 (0.68–0.88)**	**0.85 (0.75–0.97)**
Q3	29.7	340/16 385 (2.1)	**0.58 (0.50–0.66)**	**0.65 (0.56–0.74)**
Q4	43.3	368/16 385 (2.2)	**0.63 (0.55–0.72)**	**0.72 (0.63–0.83)**
Q5	69.3	387/16 385 (2.4)	**0.66 (0.58–0.75)**	**0.73 (0.64–0.84)**
*p* for trend			**<0.0001**	**<0.0001**
n-3 PUFAs				
Q1	1.09	515/16 384 (3.1)	1.00	1.00
Q2	1.46	443/16 385 (2.7)	**0.86 (0.75–0.97)**	0.90 (0.79–1.02)
Q3	1.74	388/16 385 (2.4)	**0.75 (0.65–0.85)**	**0.82 (0.71–0.94)**
Q4	2.03	367/16 385 (2.2)	**0.71 (0.62–0.81)**	**0.77 (0.67–0.89)**
Q5	2.55	414/16 385 (2.5)	**0.80 (0.70–0.91)**	**0.85 (0.74–0.97)**
*p* for trend			**<0.0001**	**0.004**

a Dietary intake during pregnancy (after learning of pregnancy). Quintile medians in g/day adjusted energy intake using the residual method.

b Covariates were adjusted for age, previous deliveries, BMI at 1 month after delivery, maternal highest educational level, annual household income, marital status at 6 months after delivery, alcohol intake at 1 month after delivery, smoking status at 1 month after delivery, physical activity during mid-late pregnancy, employment status during mid-late pregnancy for the analysis of postpartum depression at 6 months after delivery, employment status for the analysis of serious mental illness at 1 year after delivery, history of anxiety disorder, history of depression, sadness experienced during the past year, use of EPA and/or DHA supplementation, and presence of any congenital anomaly.

Values in bold are significant.

When the lowest decile was used as a reference, the fish intake results were largely the same as for the lowest quintile. Analyses determined adjusted ORs of 0.91 (95% CI 0.84–0.99), 0.86 (0.79–0.93), 0.86 (0.79–0.93), and 0.85 (0.78–0.92) at 6 months for the second, third, fourth, and fifth quintiles, respectively, and of 0.87 (0.75–1.01), 0.66 (0.56–0.78), 0.74 (0.63–0.87), and 0.75 (0.64–0.88), respectively, at 1 year. However, the results of n-3 PUFA intake using the lowest decile revealed that the risks were much lower than with the lowest quintile, with ORs of 0.84 (0.78–0.91), 0.78 (0.72–0.85), 0.73 (0.67–0.79), and 0.79 (0.73–0.86), respectively, at 6 months and of 0.83 (95% CI 0.71–0.96), 0.75 (0.64–0.88), 0.71 (0.61–0.84), and 0.78 (0.67–0.91), respectively, at 1 year.

## Discussion

In this nationwide longitudinal study, which to our knowledge is the largest of its kind, women with higher fish and/or n-3 PUFA intake showed reduced risk of postpartum depression at 6 months after delivery, with trend tests also revealing a significant linear association. Women with higher fish and/or n-3 PUFA intake also showed reduced risk of serious mental illness at 1 year after delivery, with trend tests showing a significant linear association.

In this study, the prevalence of postpartum depression at 6 months was 11.6% and the prevalence of serious mental illness at 1 year was 2.6%, which are lower than the rates determined in the prenatal period and at 1 month after delivery in our previous study (13.8% at 1 month after delivery for depression; 3.4% in the first trimester; and 3.1% in the second/third trimester for serious mental illness (Hamazaki *et al*., 2018)). These decreases over time are in line with a previous report on primiparas in Japan (Iwata *et al*., 2016). Notably, when using the lowest threshold (the lowest decile as a reference) for n-3 PUFA intake, risk was lower than when using the same threshold for fish intake. Because the present study does not include data on individual n-3 PUFAs, future work should explore thresholds for intake of individual n-3 PUFAs (e.g. *α*-linolenic acid, EPA, and DHA) or even tissue levels (e.g. erythrocytes, plasma, and serum).

We decided to comprehensively investigate whether fish and/or n-3 PUFA intake was associated with postpartum depression and serious mental illness not only because the prevalence of postpartum depression peaks at about 1 month and its symptoms become fixed and intractable at about 6 months, but also because imaging studies have reported a reduction in brain volume in pregnant women. A magnetic resonance imaging study revealed a symmetrical pattern of extensive volume reduction in gray matter (around 1%) in primiparous women throughout pregnancy, mainly affecting the anterior and posterior cortical midline and specific sections of the bilateral lateral prefrontal and temporal cortex (Hoekzema *et al*., 2017). These volume reductions largely continued, except for some recovery in the hippocampus, for another 2 years after delivery (Hoekzema *et al*., 2017). Although the authors did not discuss any involvement of PUFAs in their paper, we speculated that the volume reductions could feasibly be due, at least in part, to the loss of PUFAs, because PUFAs are abundant in the brain and especially in gray matter [about 8–10% for arachidonic acid and 9–14% for DHA (Hamazaki *et al*., 2012; Hamazaki *et al*., 2016)]. Moreover, in dams fed a DHA-deficient diet, a sufficient supply of DHA for the fetus was shown to be sourced from the maternal brain, which reduced maternal DHA levels by about 25% from pregnancy to lactation (Levant *et al*., 2006). A postmortem study of the brain from patients with major depressive disorders and from normal controls revealed that women with ⩾2 children had significantly lower DHA levels than women who had 1 child or no children, regardless of disease state (McNamara *et al*., 2007). For further analysis, it might be interesting to extend the follow-up for another year to investigate whether the association between n-3 PUFAs and risk of postpartum depression persists.

Associations were stronger for fish intake than for n-3 PUFA intake at 1-year analyses in this study; the same pattern of association was observed in our previous study on perinatal depression (Hamazaki *et al*., 2018). This is probably because some nutrients in fish other than n-3 PUFAs [i.e. vitamins (Miyake *et al*., 2015*a*), minerals (Islam *et al*., 2018), and calcium (Miyake *et al*., 2015*b*)] contributed to the reduced risk of serious mental illness. Another reason might be that data on individual n-3 PUFAs were not present in the dataset that we used in this study. In fact, dietary intake of EPA + DHA has been shown by a meta-analysis to have a more beneficial effect than total n-3 PUFAs (Grosso *et al*., 2016).

Interestingly, the ORs showed an inverted J-shaped pattern in the 6-month analysis (for n-3 PUFAs) and in the 1-year analysis (for n-3 PUFAs), similar to the U-shaped pattern observed in our previous study on perinatal depression (Hamazaki *et al*., 2018). The very first prospective study conducted in Japan by Miyake *et al*. (2006) also found the same result for intake of n-3 PUFAs (an inverted J-shaped pattern). They did not discuss this phenomenon and we do not have a clear explanation for this finding either. One plausible reason might be the n-6/n-3 ratio. Dose-ranging studies of EPA (Peet and Horrobin, 2002) and DHA (Mischoulon *et al*., 2008) revealed that the lower doses showed beneficial effects on depression but not the higher dose. The authors of the latter study speculated that an ‘optimal’ n-6/n-3 ratio exists in humans which maintains a balance between pro- and anti-inflammatory forces and that higher n-3 doses lead to an ‘overcorrection’ that dampens the antidepressant effect of n-3 PUFAs. Future studies should examine the patterns of n-6 PUFAs and the n-6/n-3 ratio according to the risk of postpartum depression using the JECS data. In addition, it is important to note that arachidonic acid is not only a precursor for the proinflammatory eicosanoids prostaglandin E2 and leukotriene B4, but also for lipoxin A4, which exerts anti-inflammatory bioactivity (Das, 2018).

To our knowledge, there have been six studies (Browne *et al*., 2006; Miyake *et al*., 2006; Golding *et al*., 2009; Strøm *et al*., 2009; da Rocha and Kac, 2012; Kobayashi *et al*., 2017) of the association between fish and/or n-3 PUFAs (or n-6/n-3 ratios) during pregnancy and the risk of postpartum depression; of these, five investigated the risk at 6 months or at longer periods (Browne *et al*., 2006; Miyake *et al*., 2006; Golding *et al*., 2009; Strøm *et al*., 2009; Kobayashi *et al*., 2017). Most found little evidence to support an association (Browne *et al*., 2006; Miyake *et al*., 2006; Strøm *et al*., 2009; Kobayashi *et al*., 2017). The one study to prospectively show an association (Golding *et al*., 2009) was originally designed to cross-sectionally investigate associations between n-3 PUFA intake and depressive symptoms at 32 weeks of gestation. That study also investigated depressive symptoms both retrospectively and prospectively and found weak associations at 18 weeks of gestation and at 8 months (but not at 2 months) after delivery, although the details were not provided in the article (Golding *et al*., 2009). These mixed and inconclusive findings may be attributable to differences in study design, sample size, duration of follow-up, participant background factors, and/or the tools used to assess depression, suggesting that further research is needed. Because a meta-analysis (Lin *et al*., 2010) reported larger differences in serum n-3 PUFA levels in studies of major depressive disorder defined according to DSM criteria than in studies not using these criteria, additional research using clinically diagnosed depression might help us to definitively determine whether there is an effect of fish or n-3 PUFAs on the risk of postpartum depression.

This is the largest study to date that investigates the association of dietary intake of fish and/or n-3 PUFA with the risk of postpartum depression at 6 months and of serious mental illness at 1 year after delivery. Because the study was conducted in 15 regions across the country, its participants can be considered representative of pregnant women in Japan. The importance of these findings is that they provide stronger evidence for a negative association of fish and/or n-3 PUFA consumption with postpartum depression and serious mental illness at 6 months and at 1 year after delivery, respectively, based on a large nationally representative sample in Japan. This is notable because even in Japan, where the consumption of fish and/or n-3 PUFAs was high compared with studies in other countries, past results have been inconsistent (Miyake *et al*., 2006; Kobayashi *et al*., 2017). This study contributes to resolving this inconsistency and improving our understanding of the benefits of fish and/or n-3 PUFA consumption in the perinatal period.

Although we used the validated and widely used K6 and EPDS screening tools, the K6 has not been validated for use in the perinatal population. The Food Frequency Questionnaire we used has not been validated for use with pregnant women either. Another limitation of this observational study is that unmeasured residual factors such as ‘health consciousness’ might have confounded the results. Also, the dataset did not contain data on the n-3 PUFA subtypes of *α*-linolenic acid, EPA, docosapentaenoic acid, or DHA. If such data had been available, we might have found a clearer relationship with depression. Finally, we cannot rule out the possibility that the exclusion of ~20 000 women based on the study criteria, while necessary, introduced some selection bias.

In conclusion, women with higher fish and/or n-3 PUFA intake showed reduced risk of postpartum depression at 6 months after delivery, with trend tests also revealing a significant linear association. These women also showed reduced risk of serious mental illness at 1 year after delivery, with trend tests revealing a significant linear association. Further investigations, particularly interventional studies, are required to confirm our findings.

## Supplementary material

For supplementary material accompanying this paper visit https://doi.org/10.1017/S0033291719002587. click here to view supplementary material

## Appendix

We would like to express our gratitude to all of the participants in this study and all of the individuals involved in data collection. We would also like to thank the following members of the JECS (Principal Investigator, Michihiro Kamijima) as of 2019: Shin Yamazaki (National Institute for Environmental Studies, Tsukuba, Japan), Yukihiro Ohya (National Center for Child Health and Development, Tokyo, Japan), Reiko Kishi (Hokkaido University, Sapporo, Japan), Nobuo Yaegashi (Tohoku University, Sendai, Japan), Koichi Hashimoto (Fukushima Medical University, Fukushima, Japan), Chisato Mori (Chiba University, Chiba, Japan), Shuichi Ito (Yokohama City University, Yokohama, Japan), Zentaro Yamagata (University of Yamanashi, Chuo, Japan), Hidekuni Inadera (University of Toyama, Toyama, Japan), Michihiro Kamijima (Nagoya City University, Nagoya, Japan), Takeo Nakayama (Kyoto University, Kyoto, Japan), Hiroyasu Iso (Osaka University, Suita, Japan), Masayuki Shima (Hyogo College of Medicine, Nishinomiya, Japan), Youichi Kurozawa (Tottori University, Yonago, Japan), Narufumi Suganuma (Kochi University, Nankoku, Japan), Koichi Kusuhara (University of Occupational and Environmental Health, Kitakyushu, Japan), and Takahiko Katoh (Kumamoto University, Kumamoto, Japan).
