# Supplementary material for: Dietary intake of fish and n-3 polyunsaturated fatty acids and risk of postpartum depression: a nationwide longitudinal study – the Japan Environment and Children's Study (JECS)
Source: Psychol Med. 2019 Sep 19;50(14):2416–24. doi: 10.1017/S0033291719002587 (PMC7610183; doi:10.1017/S0033291719002587)
Supplement: Supplementary file 1 [file S0033291719002587sup001.docx]

**Supplementary Table1.** Characteristics according to quintiles for n-3 PUFAs intake in during the pregnancy in women (n= 84,181)

|  | Quintile for n-3 PUFA intake | | | | |
| --- | --- | --- | --- | --- | --- |
|  | 1 (low) | 2 | 3 | 4 | 5 (high) |
| **Median intake of n-3 PUFAs ^a^, g/day** | 1.09 | 1.46 | 1.74 | 2.04 | 2.57 |
| **Age at delivery, years** | 30.9 | 31.2 | 31.4 | 31.6 | 31.4 |
| **Previous deliveries, n (%)** |  |  |  |  |  |
| Primipara | 7,670 (45.6) | 7,275 (43.2) | 7,163 (42.6) | 7,072 (42.0) | 7,342 (43.6) |
| Multipara | 9,166 (54.4) | 9,561 (56.8) | 9,674 (57.5) | 9,764 (58.0) | 9,494 (56.4) |
| **BMI at one month after delivery, n (%)** |  |  |  |  |  |
| <18.5 | 723 (4.3) | 749 (4.5) | 826 (4.9) | 892 (5.3) | 1,070 (6.4) |
| 18.5-<25 | 13,117 (77.9) | 13,322 (79.1) | 13,490 (80.1) | 13,522 (80.3) | 13,351 (79.3) |
| ≥25 | 2,996 (17.8) | 2,765 (16.4) | 2,521 (15.0) | 2,422 (14.4) | 2,415 (14.3) |
| **Highest educational level, n (%)** |  |  |  |  |  |
| Junior high school or high school | 6,585 (39.1) | 6,010 (35.7) | 5,566 (33.1) | 5,510 (32.7) | 5,852 (34.8) |
| Technical junior college, technical/vocational college or associate degree | 7,044 (41.8) | 7,103 (42.2) | 7,301 (43.4) | 7,276 (43.2) | 7,171 (42.6) |
| Bachelor’s degree, postgraduate degree | 3,207 (19.1) | 3,722 (22.1) | 3,970 (23.6) | 4,051 (24.1) | 3,813 (22.7) |
| **Annual household income (JPY), n (%)** |  |  |  |  |  |
| <4 million | 7,232 (43.0) | 6,744 (40.1) | 6,487 (38.5) | 6,406 (38.1) | 6,606 (39.2) |
| 4-6 million | 5,285 (31.4) | 5,573 (33.1) | 5,599 (33.3) | 5,738 (34.1) | 5,722 (34.0) |
| >6 million | 4,319 (25.7) | 4,519 (26.8) | 4,751 (28.2) | 4,692 (27.9) | 4,508 (26.8) |
| **Marital status, n (%)** |  |  |  |  |  |
| Married (including common law marriage) | 16,377 (97.3) | 16,529 (98.2) | 16,577 (98.5) | 16,552 (98.3) | 16,544 (98.3) |
| Divorced | 194 (1.2) | 126 (0.8) | 127 (0.8) | 123 (0.7) | 125 (0.7) |
| Widowed others | 265 (1.6) | 181 (1.1) | 132 (0.8) | 161 (1.0) | 167 (1.0) |
| **Alcohol intake, n (%)** |  |  |  |  |  |
| Never | 15,360 (91.2) | 15,434 (91.7) | 15,449 (91.8) | 15,433 (91.7) | 15,350 (91.2) |
| Ex-drinker | 740 (4.4) | 752 (4.5) | 756 (4.5) | 755 (4.5) | 766 (4.6) |
| 1-3 times/month | 496 (3.0) | 425 (2.5) | 448 (2.7) | 432 (2.6) | 481 (2.9) |
| ≧1 or more | 241 (1.4) | 225 (1.3) | 185 (1.1) | 216 (1.3) | 239 (1.4) |
| **Smoking status, n (%)** |  |  |  |  |  |
| Never | 9,436 (56.1) | 10,006 (59.4) | 10,205 (60.6) | 10,173 (60.4) | 10,003 (59.4) |
| Did previously but quit before learning of pregnancy | 3,796 (22.6) | 3,724 (22.1) | 3,762 (22.3) | 3,884 (23.1) | 3,826 (22.7) |
| Did previously but quit after learning of pregnancy | 2,739 (16.3) | 2,441 (14.5) | 2,304 (13.7) | 2,244 (13.3) | 2,396 (14.2) |
| Currently smoking | 865 (5.1) | 664 (4.0) | 567 (3.4) | 534 (3.2) | 611 (3.6) |
| **Median physical activity (mets∙min/day)** | 70.7 | 70.7 | 70.7 | 70.7 | 75.4 |
| **History of anxiety disorder, yes (%)** | 543 (3.2) | 494 (2.9) | 443 (2.6) | 461 (2.7) | 464 (2.8) |
| **History of depression, yes (%)** | 533 (3.2) | 508 (3.0) | 503 (3.0) | 490 (2.9) | 561 (3.3) |
| **Experiencing sadness during the past year** | 7,595 (45.1) | 7,503 (44.6) | 7,546 (44.8) | 7,369 (43.8) | 7,345 (43.6) |
| **Employed, n (%)** | 9,845 (58.5) | 9,373 (55.7) | 9,248 (54.9) | 8,924 (53.0) | 8,416 (50.0) |
| **Use of EPA and/or DHA supplementation, yes(%)** | 517 (3.1) | 476 (2.8) | 436 (2.6) | 441 (2.6) | 495 (2.9) |
| **Any congenital anomaly, n (%)** | 402 (2.4) | 352 (2.1) | 352 (2.1) | 407 (2.4) | 385 (2.3) |

^a^ Dietary intake during pregnancy (after learning of pregnancy).

BMI, body mass index
